# *Staphylococcus aureus* Multiplexes Death-Effector Deoxyribonucleosides to Neutralize Phagocytes

**DOI:** 10.3389/fimmu.2022.847171

**Published:** 2022-03-10

**Authors:** Eshraq Tantawy, Nicoletta Schwermann, Tjorven Ostermeier, Annette Garbe, Heike Bähre, Marius Vital, Volker Winstel

**Affiliations:** ^1^ Research Group Pathogenesis of Bacterial Infections, TWINCORE, Centre for Experimental and Clinical Infection Research, a Joint Venture Between the Hannover Medical School and the Helmholtz Centre for Infection Research, Hannover, Germany; ^2^ Institute of Medical Microbiology and Hospital Epidemiology, Hannover Medical School, Hannover, Germany; ^3^ Research Core Unit Metabolomics, Hannover Medical School, Hannover, Germany

**Keywords:** *Staphylococcus aureus*, immune evasion, deoxyribonucleosides, deoxyguanosine, phagocyte, apoptosis, macrophage

## Abstract

Adenosine synthase A (AdsA) is a key virulence factor of *Staphylococcus aureus*, a dangerous microbe that causes fatal diseases in humans. Together with staphylococcal nuclease, AdsA generates deoxyadenosine (dAdo) from neutrophil extracellular DNA traps thereby igniting caspase-3-dependent cell death in host immune cells that aim at penetrating infectious foci. Powered by a multi-technological approach, we here illustrate that the enzymatic activity of AdsA in abscess-mimicking microenvironments is not restricted to the biogenesis of dAdo but rather comprises excessive biosynthesis of deoxyguanosine (dGuo), a cytotoxic deoxyribonucleoside generated by *S. aureus* to eradicate macrophages of human and animal origin. Based on a genome-wide CRISPR-Cas9 knock-out screen, we further demonstrate that dGuo-induced cytotoxicity in phagocytes involves targeting of the mammalian purine salvage pathway-apoptosis axis, a signaling cascade that is concomitantly stimulated by staphylococcal dAdo. Strikingly, synchronous targeting of this route by AdsA-derived dGuo and dAdo boosts macrophage cell death, indicating that *S. aureus* multiplexes death-effector deoxyribonucleosides to maximize intra-host survival. Overall, these data provide unique insights into the cunning lifestyle of a deadly pathogen and may help to design therapeutic intervention strategies to combat multidrug-resistant staphylococci.

## Introduction

*Staphylococcus aureus* is a dangerous pathogen that persistently colonizes large segments of the human population (1, 2). *S. aureus* resides within the anterior nares of humans but also colonizes the skin surfaces, axillae, the gastrointestinal tract, and the inguinal region (2–5). While nasopharyngeal carriage of *S. aureus* is not harmful *per se*, colonized individuals, children and the elderly, or hospitalized patients with major surgery, hemodialysis, endotracheal intubation, diabetes, or immunosuppressive therapies are at elevated risk to acquire invasive disease (2, 6–8). Nonetheless, most staphylococcal infections occur in immunocompetent or otherwise healthy individuals (6, 7). Staphylococcal disease includes skin and soft tissue infections, bacteremia, sepsis, septic arthritis, and osteomyelitis (1, 6, 9). Moreover, *S. aureus* is a frequent cause of abscesses, endocarditis, ventilator-associated pneumonia, toxic-shock syndrome, or surgical wound infections (1, 6, 9). Successful treatment of these infections remains challenging and often requires administration of last-resort antibiotics as the large-scale usage of antimicrobial agents has selected for methicillin-resistant *S. aureus* (MRSA) and other multidrug-resistant clones (6, 9, 10). In addition, staphylococci frequently exchange new resistance and virulence genes by horizontal gene transfer events resulting in the emergence of new hyper-virulent variants such as community-acquired MRSA (CA-MRSA), a major cause of fulminant infections with poor or lethal clinical outcomes (6, 10, 11). Combined with the pathogen’s ability to trigger recurrent and chronic infections, hospital- and community-acquired infections caused by MRSA are still associated with high morbidity and mortality rates in many countries (9, 12, 13).

Staphylococcal invasion and proliferation in host tissues is accompanied by a massive release of bacterial products (i.e. peptidoglycan, formyl-peptides, and lipoproteins) and the secretion of cytolytic toxins that together trigger immune signaling and host cell death (14–17). Concurrently, damaged host tissues produce cytokines and chemo-attractants in response to staphylococci, or release otherwise-sequestered intracellular molecules such as nucleosomes or mitochondrially synthesized *N*-formylated peptides known to potentiate inflammation (18–21). These danger signals primarily attract neutrophils to infectious foci, providing the pathophysiological basis for the development of pyogenic abscesses, a major hallmark of staphylococcal disease (18, 19, 22). Such lesions consist of replicating staphylococcal abscess communities surrounded by broad layers of infiltrating innate immune cells of the mammalian host (22–24). Further, abscess architecture involves the formation of pathogen-immobilizing neutrophil extracellular DNA traps (NETs) as well as the deposition of structural fibrin capsules that shield healthy tissues from the disseminating pathogen (19, 22, 23, 25). However, macrophages and other phagocytes capable of eliminating staphylococci are excluded from the deeper cavity of these lesion, raising the possibility that selective maneuvers of the invading microbe are at play (26–28). In fact, emerging literature suggests that two synergistically acting enzymes of staphylococci, secreted nuclease (Nuc) and the cell surface-attached 5’-3’-nucleotidase adenosine synthase A (AdsA), impede abscess entry of macrophages thereby exacerbating pathology scores and disease outcomes in mouse models of infectious disease (26, 27). Specifically, Nuc-mediated disruption of NETs provokes the formation of deoxyadenosine monophosphate (dAMP) which is subsequently converted by AdsA into deoxyadenosine (dAdo), a potent deoxyribonucleoside that exquisitely kills macrophages during abscess formation by targeting the mammalian purine salvage pathway (26, 29). Following this strategy, *S. aureus* induces caspase-3-dependent cell death of phagocytes that aim at penetrating the deeper cavity of abscesses (26, 27). Consistent with this model, mice lacking caspase-3 expression in hematopoietic cells, including macrophages and dendritic cells, displayed increased resistance to *S. aureus* as loss of caspase-3 rendered host phagocytes resistant to staphylococcal dAdo and led to the accumulation of macrophages along with accelerated clearance of staphylococci in deep-seated abscesses, thereby underscoring the paradigm of dAdo-driven immune evasion (27). Nonetheless, deep histological profiling of abscess thin-sections not only uncovered the infiltration behavior and spatial location of specific innate immune cells within these lesions in response to staphylococcal stimuli, but also populations of necrotic immune cells that certainly release large quantities of DNA material into the abscess cavity (23, 28). Thus, staphylococcal infectious foci represent complex multicellular assemblies and particularly DNA-rich microenvironments where Nuc-mediated fragmentation of DNA strands expelled from toxin-damaged or netting immune cells may release auxiliary substrates for AdsA, ultimately questioning the biochemical repertoire of AdsA at the abscess-microbe interface.

Here, we used a multi-facetted approach to investigate the enzymatic properties of AdsA in a DNA-rich microenvironment that aims at simulating staphylococcal abscesses. We show that the activity of AdsA is not limited to the formation of dAdo, but rather involves excessive biogenesis of deoxyguanosine (dGuo). Of note, dGuo exhibits cytotoxic properties and is generated by *S. aureus* to eliminate host phagocytes by targeting the mammalian purine-salvage pathway-apoptosis axis. As this cell death signaling cascade is co-stimulated by staphylococcal dAdo, we further reveal that simultaneous targeting of this pathway potentiates killing of macrophages. These results suggest that a coordinated biogenesis of purine deoxyribonucleosides by AdsA may tune staphylococcal survival within infectious foci and hostile host environments.

## Material and Methods

### Bacterial Strains

All bacterial strains used in this study are listed in **Table 1**. Bacteria were grown in lysogeny broth (LB) or tryptic soy broth (TSB) at permissive temperatures. Media were supplemented with appropriate antibiotics (ampicillin 100 µg/ml; chloramphenicol 10 µg/ml).

**Table 1:** Bacterial strains and cell lines used in this study.

Bacterial strain or cell line	Description	Reference
*E. coli* Stbl3	Host strain for lentiviral vector constructs	Thermo Fisher
*E. coli* DC10B	Δ*dcm* (DH10B background); Dam methylation only	(30)
*E. coli* DC10B pBASE6-*adsA*	DC10B bearing pBASE6-*adsA* knock-out plasmid	This study
*E. coli* BL21 (DE3) pGEX-2T-*adsA*	BL21 bearing pGEX-2T-*adsA* expression plasmid	(31)
*E. coli* DC10B pRB473-*adsA*	DC10B bearing pRB473-*adsA* complementation plasmid	This study
*S. aureus* Newman	Clinical isolate	(32)
*S. aureus* Newman Δ*adsA*	Newman Δ*adsA*	This study
*S. aureus* Newman Δ*adsA* pRB473-*adsA*	Newman Δ*adsA* complemented with pRB473-*adsA*	This study
U937	U937 cell line, ATCC^®^ CRL-1593.2™	ATCC
U937 *SLC29A1* ^-/-^	U937, bi-allelic deletion in *SLC29A1*	(29)
U937 *DCK* ^-/-^	U937, bi-allelic deletion in *DCK*	(29)
U937 *CASP3* ^-/-^	U937, bi-allelic deletion in *CASP3*	(27)
RAW264.7	RAW264.7 cell line, ATCC^®^ TIB-71™	ATCC
HEK293FT	HEK293FT cell line	Thermo Fisher
HL-60	HL-60 cell line, ACC-3	DSMZ

### Cell Culture

HEK293FT cells were obtained from Thermo Fisher and grown in DMEM medium (Gibco) supplemented 10% fetal bovine serum, 0.1 mM MEM non-essential amino acids, 6 mM L-glutamine, 1 mM sodium pyruvate, and 500 µg/ml Geneticin according to the manufacturer’s instructions. U937 cells were purchased from American Type Culture Collection (ATCC) and grown in Roswell Park Memorial Institute (RPMI) 1640 medium (Gibco) supplemented with 10% heat-inactivated fetal bovine serum (hi-FBS) according to the manufacturer’s instructions. RPMI 1640 medium containing 10% hi-FBS was further used to cultivate HL-60 cells. HL-60 cells were differentiated into neutrophil-like cells in the same medium supplemented with 1.2% dimethyl sulfoxide (DMSO) for 3 days. RAW264.7 cells were obtained from ATCC and grown in Dulbecco’s Modified Eagle’s Medium (DMEM) supplemented with 10% hi-FBS according to the manufacturer’s instructions. Cells were grown at 37°C under 5% CO_2_. All mammalian cell lines used in this study are listed in **Table 1**.

### Molecular Genetics

*S. aureus adsA* was deleted by using pBASE6 and a published protocol (33). Briefly, the *adsA* gene flanking regions were amplified *via* PCR from *S. aureus* Newman genomic DNA and combined *via* overlap PCR using the primers listed in **Table 2**. The purified PCR product was cloned into linearized pBASE6 plasmid at the BglII and SacI restriction sites resulting in pBASE6-*adsA*. This plasmid was isolated from *Escherichia coli* DC10B cells (30) and transferred to *S. aureus* Newman *via* electroporation as described elsewhere (34). pBASE6-*adsA* was integrated into the genome at a permissive temperature of 43°C and in the presence of 10 µg/ml chloramphenicol. Following integration, a counterselection step was performed at 30°C using anhydrotetracycline (0.2 µg/ml). Resulting clones were streaked onto tryptic soy agar (TSA) plates with or without chloramphenicol (10 µg/ml) and screened for plasmid loss. Chloramphenicol-sensitive colonies were screened *via* PCR and Sanger sequencing to confirm gene deletion. For complementation studies, the coding sequence of the *S. aureus adsA* gene including its native promoter was amplified *via* PCR from *S. aureus* Newman genomic DNA, purified, and cloned into the previously described shuttle plasmid pRB473 at the SacI/EcoRI restriction sites (35). Primers are listed in **Table 2**. The resulting complementation plasmid pRB473-*adsA* was transferred to the *S. aureus* Newman *adsA* knock-out strain *via* electroporation as described before (34). Construction of the pGEX-2T plasmid required for expression of *S. aureus* AdsA in *E. coli* BL21 is described elsewhere (31).

**Table 2:** Oligonucleotides used to generate knock-out and complementation plasmids.

Primer	Sequence	Application	Reference
F1-adsA-up	ATCGAGCTCATTGACCGTAATATTCAAGATTTAAATGGTATTTT	pBASE6-*adsA*	This study
F1-adsA-dn	CCATTAACTGTCATACACCAATATATTATCATAAT	pBASE6-*adsA*	This study
F2-adsA-up	TATGACAGTTAATGGAATATATTGAAAATAATACTACTGTATTTCTTAAATAAGA	pBASE6-*adsA*	This study
F2-adsA-dn	GAGAAGATCTTTAGCTACTACTTAAGTTATATGCGCAATT	pBASE6-*adsA*	This study
473-adsA-up	ATCGAGCTCGACTTGGCAGGCAATTGAAAAGAA AGAT	pRB473-*adsA*	This study
473-adsA-dn	GAGAGAATTCTTAGCTAGCTTTTCTACGTCGTACAGT	pRB473-*adsA*	This study

### Isolation of Primary Cells

Human primary polymorphonuclear cells (PMNs or neutrophils) were isolated by a standard density gradient separation method as previously described (36). Peripheral blood mononuclear cells (PBMCs) were isolated from heparinized blood by density gradient centrifugation on Ficoll-Paque (PAN Biotech) according to standard laboratory protocols. Human primary monocytes were isolated from PBMCs by using magnetic nanobeads and the MojoSort™ Human CD14 Selection Kit (BioLegend) according to the manufacturer’s instructions. Following isolation, purified primary CD14^+^ monocytes were resuspended in RPMI 1640 medium supplemented with 10% hi-FBS and 1% penicillin‐streptomycin for cytotoxicity experiments or subsequently differentiated into human monocyte-derived macrophages (hMDM) in the same medium supplemented with 50 ng/ml of human macrophage colony stimulating factor (hM-CSF; Sigma). hMDM were used at day 7 post differentiation for cytotoxicity experiments. Murine bone marrow-derived macrophages (BMDM) were isolated as described earlier, with minor modifications (27). In brief, female C57BL/6 mice were purchased from Janvier Laboratories and kept under specific pathogen-free conditions in our central mouse facility (TWINCORE, Center for Experimental and Clinical Infection Research, Hannover, Germany). Mice were killed and the femur and tibia were removed according to standard laboratory protocols. Subsequently, bones were sterilized with 70% ethanol, and washed with sterile phosphate-buffered saline (PBS). Next, the ends of the bones were removed to flush out the bone marrow with RPMI 1640 containing 10% hi-FBS and 1% penicillin-streptomycin. The bone marrow was carefully resuspended and passed through a nylon cell strainer (BD; 40 μm) to remove unwanted tissue and cellular debris. The resulting cell suspension was centrifuged for 10 min at 200 x g and 4°C. Following centrifugation, the cell pellet was resuspended in red blood cell (RBC) lysis buffer (Roche) and incubated for 5 min at RT to lyse RBC according to the manufacturer’s instructions. Next, the cell suspension was centrifuged (10 min, 4°C, 200 × g) to obtain a RBC-free cell pellet which was resuspended in BMDM medium (RPMI 1640 containing 10% hi-FBS, 1% penicillin-streptomycin, and 50 ng/ml of mouse macrophage colony-stimulating factor (Genscript)). Cells were then seeded into tissue culture treated dishes to deplete bone marrow cells from fibroblasts. At day 1 post extraction, suspension bone marrow cells were collected *via* centrifugation (10 min, 4°C, 200 × g). Finally, cells were enumerated by using a hemocytometer, adjusted to 6.0 x 10^5^ cells/ml in BMDM medium, and re-seeded into bacteriological dishes. At day 4 post extraction, cells were incubated with an additional 10 ml of BMDM medium. BMDM were used at days 7 post extraction.

### Protein Purification

A recombinant and glutathione S-transferase (GST)-tagged variant of *S. aureus* AdsA (rAdsA) was expressed in *E. coli* BL21 using the pGEX-2T plasmid (GE Healthcare). Proteins were purified as described elsewhere using glutathione S-transferase affinity chromatography (31). The N-terminal GST tag was removed *via* thrombin cleavage. Thrombin was removed from the protein sample by using benzamidine sepharose beads according to the manufacturer’s instructions (GE Healthcare). Purified rAdsA was analyzed by Coomassie-stained SDS-PAGE according to standard laboratory protocols.

### Chromatography Methods

Formation of dGuo was analyzed using a thin-layer chromatography (TLC) protocol described elsewhere (37). Briefly, dGMP (final conc. 1.19 mM) was mixed with rAdsA (2.5 µg/µl) and incubated in reaction buffer (30 mM Tris-HCl, pH 7.5; 1.5 mM MgCl_2_; 1.5 mM MnCl_2_) for 16 h at 37°C. Controls lacked dGMP or rAdsA. Following incubation, reaction products along with standards were directly applied to TLC sheets (SIL G, Macherey-Nagel) which were developed in the ascending direction at room temperature by using a water/isopropanol/ammonium bicarbonate mixture (25%:75%:0.2 M). The migratory positions of dGuo and dGMP were identified with pure dGuo and dGMP standards (Sigma) that were visualized under UV light at 254 nm. rAdsA-derived dGuo was further analyzed and quantified by LC-MS/MS analytics as described before (38). Briefly, rAdsA (1.4 µg/µl) was incubated with dGMP at 37°C in reaction buffer as described above. Controls lacked dGMP or rAdsA. Following incubation, all reactions were terminated by using EDTA (final conc. 50 mM). Next, reversed phase chromatographic separation was performed using a Shimadzu HPLC-system (Shimadzu, Duisburg, Germany) consisting of two HPLC-Pumps (LC-30AD), a temperature controlled autosampler (SIL-30AC), a degasser (DGU-20A5), an oven (CTO-20AC), and a control unit (CBM-20A). A Hypercarb (30 x 4.6 mm; 5 µm; Thermo Scientific, Waltham, Massachusetts, USA) connected to a C18 security guard (Phenomenex, Aschaffenburg, Germany) along with a 0.5 µm column saver were used. The column was maintained at 50°C. Solvent A was 10 mM ammonium acetate (pH 10; adjusted with 25% NH_3_). Solvent B was acetonitrile. For analyte separation, a gradient was applied: 0 to 8 min, 4 to 60% B; 8 to 12 min 4% B. The flow rate was kept at 600 µL/min. Analysis of target substances was carried out by a tandem mass spectrometer (5500QTRAP; Sciex, Framingham, Massachusetts) equipped with an electrospray ionization source (ESI), operating in positive ionization mode and using an ion spray voltage of 4500 V. Further ESI parameters were: curtain gas (CUR): 30 psi, collision gas (CAD): 9, temperature: 600°C, gas 1: 60 psi and gas 2: 75 psi, respectively. For selected-reaction monitoring (SRM), the following mass transitions were used: dGMP: *m/z* 348→ 152 (quantifier), *m/z* 348 → 135 (identifier) and dGuo: *m/z* 268 → 152 (quantifier), *m/z* 268 → 135 (identifier). The following mass transitions were used for the internal standards: tenofovir: *m/z* 288 → 176. Control of LC and the mass spectrometer as well as data sampling was performed by Analyst software (version 1.7, Sciex). For quantification, calibration curves were created by plotting peak area ratios of the target analyte, and the internal standard versus the nominal concentration of the calibrators. The calibration curve was calculated using quadratic regression and 1/x weighing.

### Biochemical Assays

AdsA-mediated production of deoxyribonucleosides in DNA-rich microenvironments was determined as described elsewhere with minor modifications (26). In brief, nuclease-derived thymus DNA digests were treated with rAdsA (2.9 µg/µl) in reaction buffer (190.3 mM Tris, pH 7.4; 1.77 mM MgCl_2_; 1.77 mM CaCl_2_; 0.64 mM MnCl_2_) for 16 h at 37°C. Controls lacked rAdsA or DNA. All reaction products were analyzed *via* TLC as described above. To analyze *S. aureus*-mediated hydrolysis of dGMP, the *S. aureus* Newman strain panel was grown in TSB to the early log phase and washed twice in reaction buffer (30 mM Tris-HCl, pH 7.4; 2.0 mM MnCl_2_; 2.0 mM MgCl_2_). Each bacterial strain (1.0 × 10^7^ CFU) was then incubated with dGMP (final conc. 5 mM) in reaction buffer for 90 min at 37°C. Controls included reaction buffer only or lacked bacteria or dGMP. Next, samples were centrifuged for 10 min at 16,000 x g. Hydrolysis of dGMP and the associated release of inorganic phosphate in resulting culture supernatants was analyzed using a colorimetric phosphate detection kit according to the manufacturer’s instructions (Abcam). AdsA-dependent formation of dGuo was further analyzed by using *S. aureus*-derived cell wall extracts using a method described elsewhere (31, 38). Briefly, *S. aureus* overnight cultures were diluted 1:100 in TSB medium and grown to an optical density (600 nm) of 1.0. Staphylococci were washed twice in sterile PBS, and 3 ml of the resulting suspension were centrifuged and suspended in 100 µl of TSM buffer (50 mM Tris-HCl, pH 7.5; 0.5 M sucrose; 10 mM MgCl_2_) containing lysostaphin (70 µg/ml). Reactions were incubated for 30 min at 37°C and centrifuged at 9,000 x g to obtain released cell surface proteins. Fifteen microliters of the resulting cell wall extracts were incubated with 3 µl dGMP (stock solution: 10 mM) for 30 min at 37°C. Samples were applied to TLC plates and analyzed as described above. Individual dGuo spots of independent biological replicates were quantified by using the ImageJ processing package Fiji (39).

### Cytotoxicity Assays

To assess dGuo-mediated cytotoxicity, 4.0 × 10^5^ U937 cells per well were seeded in a 24-well plate and incubated for 48 h at 37°C under 5% CO_2_ in RPMI growth medium supplemented with 160 nM phorbol 12-myristate 13-acetate (PMA). Resulting U937-derived macrophages were washed once and incubated in RPMI growth medium lacking PMA for additional 24 h. Similarly, 2.0 x 10^5^ RAW264.7 cells or 4.0 x 10^5^ BMDM per well were seeded in 24-well plates and incubated for 24 h at 37°C under 5% CO_2_ in corresponding growth media. Following incubation, growth media were replaced by normal growth medium containing various concentrations of dGuo (160 µM for U937-derived macrophages and BMDM; 320 µM for RAW264.7-derived macrophages). Macrophages were further incubated for different time periods at 37°C under 5% CO_2_ as indicated in figure legends. Where indicated, cells were also exposed to the hENT1 inhibitor dipyridamole or nitrobenzylthioinosine (NBTI) (both Sigma) two hours prior treatment with dGuo, or were simultaneously treated with a mixture of dGuo and dAdo as indicated in the figure legends. Phagocytes were detached by using trypsin-EDTA solution (U937-derived macrophages; BMDM) or a cell scraper (RAW264.7-derived macrophages). Dead cells were stained with trypan blue and counted by using a microscope to calculate killing efficiency. To analyze dGuo-mediated cytotoxicity in neutrophil-like cells or primary human neutrophils (PMNs), 2.0 x 10^5^ DMSO-differentiated HL-60 cells (nHL-60) or 2.0 x 10^5^ PMNs per well were seeded in 24-well plates and incubated for 24 h at 37°C under 5% CO_2_ in standard RPMI growth medium supplemented with 160 µM dGuo. Similarly, primary CD14^+^ monocytes or hMDM were exposed to dGuo (160 µM for CD14^+^ monocytes; 320 µM for hMDM) in corresponding growth media for 24 h at 37°C under 5% CO_2_. Following incubation, hMDM were detached by using trypsin-EDTA solution. Viability of nHL-60 cells, PMNs, CD14^+^ monocytes or hMDM was recorded *via* trypan blue staining and microscopy as described above.

Cytotoxicity mediated by *S. aureus*-derived dGuo was analyzed by incubating the *S. aureus* Newman strain panel in the presence or absence of dGMP and subsequent incubation with U937-derived macrophages or RAW264.7 cells. In brief, the indicated *S. aureus* strains were grown overnight at 37°C in TSB, diluted 1:100 in fresh TSB medium and grown at 37°C to 1.5 x 10^8^ CFU/ml. Staphylococci were centrifuged (10 min, RT, 8,000 x g), washed twice in sterile wash buffer (50 mM Tris-HCl; pH 7.5), and adjusted to 3.2 x 10^8^ CFU/ml. 8.0 x 10^7^ CFU were incubated in reaction buffer (30 mM Tris-HCl, pH 7.5; 2 mM MgCl_2_) containing dGMP (final conc. 5 mM) for 90 min at 37°C. For experiments with RAW264.7 cells, the final concentration of dGMP was 10 mM. Controls lacked dGMP or bacteria or included the *S. aureus adsA* mutant, which cannot synthesize dGuo. Subsequently, bacteria were removed by a brief centrifugation and filtration step. 300 µl of the filter-sterilized supernatants were mixed with 700 µl of RPMI growth medium and incubated with U937-derived macrophages (24 h) or RAW264.7 cells (48 h) at 37°C under 5% CO_2_. Where indicated, staphylococci were also incubated with 5 mM of dGMP or dAMP alone or in combination. To calculate killing efficiency, cells were collected and stained with trypan blue as described above. To analyze dGuo-mediated cytotoxicity using recombinant AdsA, dGMP was also incubated with rAdsA as described above (see TLC section). Controls lacked dGMP or rAdsA. All resulting reaction products were filter-sterilized, added to U937-derived macrophages or RAW264.7 cells, and incubated at 37°C under 5% CO_2_ for 24 h. Following incubation, cells were collected and stained with trypan blue as described before.

### Genome-Wide CRISPR-Cas9 Screen

The genome-wide CRISPR-Cas9 screen was performed as described earlier, with minor modifications (29). Briefly, the human CRISPR-Cas9 GeCKO v2 library A was a gift from Feng Zhang (40, 41), which was purchased from Addgene (Cambridge, MA, USA). The CRISPR-Cas9 GeCKO v2 plasmid library was amplified in *E. coli* Stbl3 cells and used to produce lentiviral particles by using the Vira power kit (Thermo Fisher) according to the manufacturer’s instructions. Viral particles were harvested from HEK293FT culture supernatants 48-72 h post infection and concentrated by using Lenti-X Concentrator (Clontech). Lentiviral particles were suspended in DMEM supplemented with 10% FBS and 1% bovine serum albumin, and stored at -80°C until usage. Next, the pooled library was transduced into U937 cells *via* spinfection (1,000 x g for 2 h at room temperature) in the presence of 8 µg/ml polybrene (Sigma) at a multiplicity of infection (MOI) of approximately 0.3 as described elsewhere (29). Following spinfection, cell pellets were carefully resuspended in fresh RPMI 1640 medium containing 10% hi-FBS, incubated for 48 h, and then selected with 2.5 µg/ml puromycin for 7 days to complete gene editing. Next, 5.0 x 10^7^ puromycin-resistant U937 cells per condition were treated with dGuo (42.5 µM) or, as a control, left untreated. During this procedure, cells were passaged every 3-4 days, and continuously grown in the presence or absence of dGuo. On day 42 post intoxication, the screen was terminated, and the genomic DNA from 3.0 x 10^7^ cells was isolated by using the Blood & Cell Culture DNA Midi Kit (Qiagen, Hilden, Germany) and stored at -20°C.

### Next Generation Sequencing and Bioinformatics

DNA samples obtained from the CRISPR-Cas9 GeCKO screen were used to prepare a sgRNA library by a two-step PCR as described before (40, 41). The first PCR was used to amplify the sgRNA-containing cassette. Resulting PCR products were subjected to a second PCR using a primer pair encoding a unique 8-bp barcode required for multiplexing along with a stagger sequence to increase library complexity as described elsewhere: http://sanjanalab.org/lib.html (40, 41). Purified PCR products of the second PCR were pooled, diluted, and mixed with 10% PhiX, and sequenced with a MiSeq machine using a Micro Kit V2 flowcell (300 cycles). The raw sequencing data were processed and analyzed using the CRI CRISPR-Cas9 library screen pipelines according to MAGeCK v0.5.8 (42). In brief, sequencing reads were first de-multiplexed by using the barcode in the reverse primer and processed by Cutadapt v2.10 (43) to remove potential adapter sequences from the beginning of sgRNA priming site primers. Read counts of sgRNAs for each sample were quantified by applying MAGeCK’s *count* command. Subsequently, samples were compared using MAGeCK’s *test* command and genes were ranked according to their score. Visualization of results were done in R v4.1.0 using ggplot2 (44).

### Immunoblotting

To analyze dGuo-dependent activation of caspase-3, U937-derived macrophages, RAW264.7 cells, or BMDM were exposed to various concentrations of dGuo (80 µM for U937-derived macrophages; 320 µM for RAW264.7-derived macrophages and BMDM) or left untreated. Cells were incubated for 24 h at 37°C under 5% CO_2_ as indicated in the figure legends. Next, macrophages were detached using trypsin-EDTA solution (U937-derived macrophages), a cell scraper (RAW264.7 cells), or accutase solution (Gibco) (BMDM). Subsequently, cells were washed twice in ice-cold 1 × PBS and lysed in pre-chilled lysis buffer (50 mM HEPES, pH 7.4; 5 mM CHAPS; 5 mM DTT). During lysis, cells were kept on ice. Following lysis, cell lysates were centrifuged for 10 min at 18,000 × g and 4°C. Supernatants were mixed with sodium dodecyl sulfate-polyacrylamide gel (SDS-PAGE) loading buffer and boiled for 10 min at 95°C. Proteins were separated *via* SDS-PAGE (12%) and transferred onto PVDF membranes for immunoblot analysis with the following rabbit primary antibodies: α-Caspase-3 (α-CASP3, 9662, Cell Signaling) and α-GAPDH (ab181602, Abcam, loading control). Immunoreactive signals were revealed with a secondary antibody conjugated to horseradish peroxidase (α-rabbit IgG, 7074, Cell Signaling); horseradish peroxidase activity was detected with enhanced chemiluminescent (ECL) substrate (Thermo Fisher).

### Determination of Caspase-3 Activity

Caspase-3 activity in dGuo-exposed cells was analyzed using a colorimetric caspase-3 detection kit (Sigma). In brief, U937-derived macrophages were exposed to dGuo (160 µM) or left untreated and incubated for the indicated time periods at 37°C under 5% CO_2_. Where indicated, cells were also treated with 160 µM of dGuo or dAdo, or received a combination of both. Next, macrophages were detached using trypsin-EDTA solution and washed twice in pre-chilled 1× PBS. Subsequently, cells were lysed on ice for 20 min in ice-cold lysis buffer (Sigma kit). Following incubation, cell lysates were centrifuged (18,000 × g for 10 min, 4°C). Resulting supernatants were incubated with the Ac-DEVD-pNA substrate of caspase-3 according to the manufacturer’s instructions. Caspase-3 activity was determined based on the amount of released pNA which can be detected at 405 nm.

### FITC-Annexin-V/PI Staining

FITC-annexin-V/PI staining of U937-derived macrophages exposed to dGuo (160 µM) was performed by using the FITC-annexin-V Apoptosis Detection Kit I (BD Biosciences) according to the manufacturer’s instructions. Stained cells were either analyzed *via* flow cytometry and the FlowJo software (BD Life Sciences) or by immunofluorescence microscopy according to standard laboratory protocols.

### Ethics Statement

Human blood samples were obtained from anonymous, consenting healthy donors from the Hannover Medical School blood donation center. The Hannover Medical School blood donation center obtained written informed consent from all participants involved in the study. These studies were reviewed and approved by the medical ethics committee of Hannover Medical School (Hannover, Germany) under the permission number 8831_BO_K_2019. Scientific use of animals was carried out under approved animal care and use protocols which are granted by the state veterinary authorities and overseen by the internal animal care and use committee (IACUC).

### Statistical Analysis

Statistical analysis was performed using GraphPad Prism (GraphPad Software, Inc., La Jolla, USA). Statistically significant differences were calculated by using statistical methods as indicated. *P* values < 0.05 were considered significant.

## Results

### A Multi-Technological Approach Reveals the Biosynthesis of Deoxyguanosine by Staphylococcal AdsA

**Figure 1:**
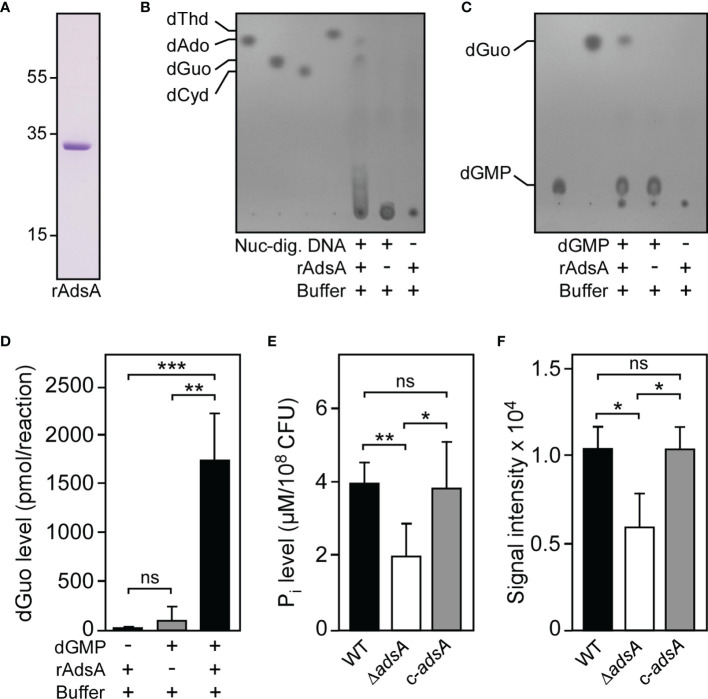
*S. aureus* deploys AdsA to synthesize deoxyguanosine. **(A)** SDS-PAGE analysis of purified rAdsA. Numbers to the left of the SDS-PAGE indicate the migration of molecular weight markers in kilodaltons. **(B, C)** Detection of dGuo formation by rAdsA. rAdsA was incubated with Nuc-digested DNA **(B)** or dGMP **(C)** at 37°C. Controls lacking Nuc-digested DNA, dGMP, or rAdsA are indicated (+ and – symbols). Reaction products and the formation of dGuo were analyzed *via* TLC. The migratory positions of single deoxyribonucleosides or dGMP were identified using pure standards. Representative images are shown. **(D)** Detection and quantification of rAdsA-derived dGuo by LC-MS/MS. rAdsA was incubated with dGMP at 37°C. Controls lacking dGMP or rAdsA are indicated (+ and – symbols). Reaction products and the formation of dGuo were analyzed and quantified *via* LC-MS/MS. **(E)** *S. aureus*-dependent hydrolysis of dGMP. The ability of *S. aureus* to hydrolyze dGMP in an AdsA-dependent manner was evaluated by assessing the release of inorganic phosphate (P_i_) using a malachite green-based colorimetric assay. Wild-type *S. aureus* Newman (WT) or the mutant lacking *adsA* (Δ*adsA*) along with the complemented *adsA* variant (c-*adsA*) are indicated. **(F)** Analysis and quantification of *S. aureus*-dependent formation of dGuo. Lysostaphin-generated cell wall extracts of indicated strains were incubated with dGMP at 37°C and analyzed *via* TLC as described above. Individual dGuo spots of independent replicates were quantified by using ImageJ. Data are the mean (± standard deviation [SD]) values from at least three independent determinations. Statistically significant differences were analyzed with one-way analysis of variance (ANOVA) and Tukey’s multiple-comparison test **(D–F)**; ns, not significant (*P* ≥ 0.05); *P < 0.05; **P < 0.01; ***P < 0.001.

Earlier work demonstrated that staphylococcal nuclease serves as an extracellular phosphodiesterase that liberates 3’-phosphonucleotides and dinucleotides from DNA molecules (45–47). Since this enzymatic feature will certainly foster the accumulation of all canonical deoxyribonucleotides during *S. aureus* infection, we initially wondered whether nuclease-driven degradation of host DNA along with the subsequent activity of staphylococcal AdsA may lead to the generation of a cocktail of bioactive deoxyribonucleosides within DNA-rich microenvironments. To test this possibility, we incubated a purified, recombinant form of *S. aureus* AdsA (31) (hereafter termed rAdsA) with nuclease-digested DNA and screened reaction products for the presence or absence of deoxyribonucleosides *via* thin-layer chromatography (TLC) (**Figures 1A, B**). Notably, this approach revealed that the synergistic activity of *S. aureus* nuclease and AdsA not only resulted in the formation of dAdo, but also in the biogenesis of a second, highly abundant deoxyribonucleoside species (**Figure 1B**). Based on purified deoxyribonucleosides, we speculated that the novel compound may represent deoxyguanosine (dGuo) (**Figure 1B**). To confirm this assumption, we took advantage of a more defined experimental condition where rAdsA was mixed solely with purified dGMP and assayed *via* TLC. The analysis revealed that rAdsA efficiently hydrolyzed dGMP as dGuo exclusively accumulated in enzymatic reactions that contained dGMP and the protein (**Figure 1C**). These results and the rAdsA-dependent biosynthesis of dGuo were further validated by using high performance liquid chromatography-coupled tandem mass spectrometry (HPLC-MS/MS), which proofed the rAdsA-catalyzed hydrolysis of dGMP and associated formation of dGuo (**Figure 1D**). Together, these initial data suggest that staphylococcal AdsA is capable of hydrolyzing dGMP for subsequent formation of dGuo.

To investigate whether the AdsA-dependent hydrolysis of dGMP and coupled biosynthesis of dGuo can be recapitulated by using viable staphylococci, we generated a *S. aureus* Newman *adsA* mutant panel and made use of a previously described experimental approach (38). Specifically, we incubated wild-type *S. aureus* Newman and the appropriate *adsA* mutant with purified dGMP and assayed for the release of inorganic phosphate using a commercially available malachite green-based colorimetric assay. Compared to wild-type bacteria, lack of *adsA* caused a significant reduction of measurable inorganic phosphate, demonstrating that the *S. aureus* Newman *adsA* variant lost the capacity to hydrolyze dGMP (**Figure 1E**). Of note, genetic complementation by using a plasmid-borne copy of *adsA* (pRB473-*adsA*) restored the ability of the *adsA* mutant to hydrolyze dGMP to levels comparable to those seen in the parental strain, suggesting that *S. aureus* deploys AdsA to synthesize dGuo (**Figure 1E**). Moreover, we digested peptidoglycan of the *S. aureus* Newman *adsA* mutant panel with lysostaphin to obtain cell wall extracts that were incubated with dGMP for subsequent analysis by TLC. Cell wall extracts derived from the *adsA* mutant displayed markedly reduced nucleotidase activity as revealed by quantification of dGuo-specific signals following TLC (**Figure 1F**). These effects could also be restored to wild-type levels when the *adsA* mutant was transformed with pRB473-*adsA* further indicating that *S. aureus* requires AdsA to synthesize dGuo (**Figure 1F**). Thus, *S. aureus* deploys AdsA not only for the generation of dAdo but also for excessive biogenesis of dGuo.

### *S. aureus* Exploits the Cytotoxic Properties of Deoxyguanosine to Promote Immune Cell Death

**Figure 2:**
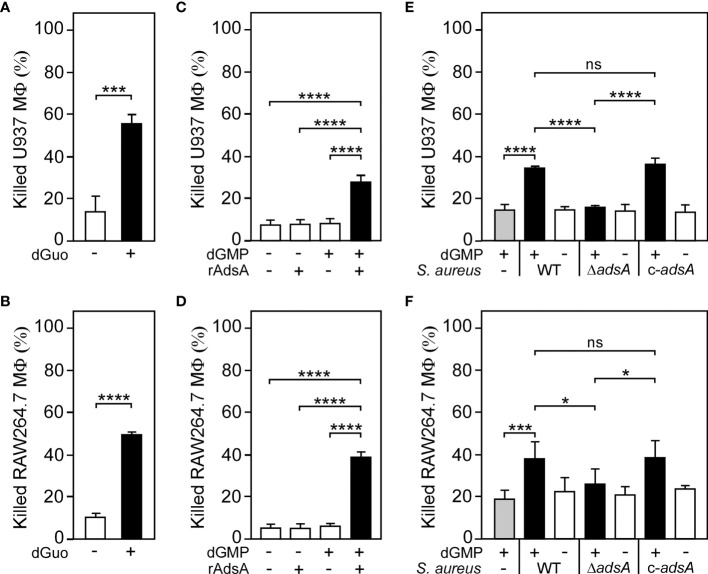
*S. aureus* generates deoxyguanosine to kill phagocytes. **(A, B)** Survival rates of human U937-derived macrophages (MФ) **(A)** or murine RAW264.7 MФ **(B)** exposed to dGuo (+) or left untreated (-). **(C, D)** Survival rates of U937-derived MΦ **(C)** or murine RAW264.7 MФ **(D)** exposed to rAdsA-derived dGuo. rAdsA was incubated with dGMP and reaction products containing dGuo were used to kill phagocytes. Controls lacked rAdsA or dGMP or included reaction buffer only as indicated with + and − symbols. **(E, F)** Survival of U937-derived MΦ **(E)** or murine RAW264.7 MФ **(F)** after treatment with culture medium (RPMI 1640) that had been conditioned by incubation with either wild-type (WT) *S. aureus* Newman, its *adsA* mutant (Δ*adsA*), or the complemented *adsA* variant (c-*adsA*) in the presence (black bars) or absence (white bars) of dGMP. Controls included culture medium that had been conditioned by incubation with dGMP only (gray bars). 160 µM **(A)** or 320 µM **(B)** of dGuo was used to treat the cells. Cell survival rates were analyzed 24 h **(A, C–E)** or 48 h **(B, F)** post-treatment. Data are the mean (± standard deviation [SD]) values from at least three independent determinations. Statistically significant differences were analyzed by a two-tailed Student’s t-test **(A, B)** or with one-way analysis of variance (ANOVA) and Tukey’s multiple-comparison test **(C–F)**; ns, not significant (*P* ≥ 0.05); *P < 0.05; ***P < 0.001; ****P < 0.0001.

Having demonstrated that *S. aureus* is capable of generating dGuo in an AdsA-dependent manner, we next sought to uncover the biological function of dGuo during staphylococcal infections. Since *S. aureus*-derived dAdo exhibits immunomodulatory attributes and triggers macrophage exclusion from infectious foci (26, 27), it seemed plausible to us at this stage that staphylococci may generate dGuo to manipulate phagocytes that play a significant role during staphylococcal infections and abscess formation. Initially, we exposed human U937-derived macrophages to dGuo and recorded survival rates 24-hours post intoxication. dGuo killed U937-derived macrophages, as revealed by conventional trypan blue exclusion assays and light microscopy (**Figure 2A**). Moreover, we observed that dGuo provoked cell death in primary human CD14^+^ monocytes and human monocyte-derived macrophages (hMDM) (**Supplementary Figures 1A, B**). Similar findings were obtained for murine RAW264.7 cells or primary bone marrow-derived macrophages (BMDM) indicating that dGuo also exhibits cytotoxic properties toward immune cells of animal origin (**Figure 2B** and **Supplementary Figure 1C**). However, HL-60-derived neutrophil-like cells (nHL-60) or primary blood neutrophils were less susceptible to dGuo-induced cytotoxicity suggesting that this deoxyribonucleoside may selectively target a specific subset of phagocytes during infection (**Supplementary Figures 2A, B**). Collectively, these data demonstrate that dGuo predominantly triggers cell death of macrophages.

To evaluate the relevance of AdsA-mediated biosynthesis of dGuo during *S. aureus* pathogenesis, we incubated purified rAdsA with or without dGMP as described above and added the resulting and filter-sterilized reaction products to U937-derived macrophages. Of note, rAdsA/dGMP-derived and dGuo-containing reaction products, but not samples that lacked dGMP or rAdsA, provoked cell death of phagocytes in this approach (**Figure 2C**). Likewise, rAdsA-derived dGuo killed RAW264.7 macrophages in a fashion similar to U937-derived macrophages, thereby underscoring the cytotoxic potential of this deoxyribonucleoside (**Figure 2D**). To further delineate the role of *S. aureus*-derived dGuo in killing phagocytes, we refined a previously described approach (26, 27, 29) and incubated the *S. aureus* Newman *adsA* mutant panel with or without dGMP to obtain conditioned culture media, which were filter-sterilized and transferred to human U937-derived macrophages. Notably, killing of U937-derived macrophages required both *S. aureus* expressing *adsA* and dGMP as only *S. aureus* Newman wild type and dGMP conditioned culture media promoted cell death in these experiments (**Figure 2E**). Moreover, genetic complementation restored the cytotoxicity profile of the *adsA* mutant when incubated with dGMP further indicating that *S. aureus* triggers cell death of host immune cells by synthesizing dGuo (**Figure 2E**). Lastly, RAW264.7 macrophages were also susceptible to *S. aureus*-derived dGuo, confirming that staphylococci utilize AdsA-mediated biosynthesis of dGuo to eliminate phagocytes of mammalian origin (**Figure 2F**).

### Genome-Scale CRISPR-Cas9 Screening Identifies Host Factors Required for Deoxyguanosine-Mediated Cytotoxicity

**Figure 3:**
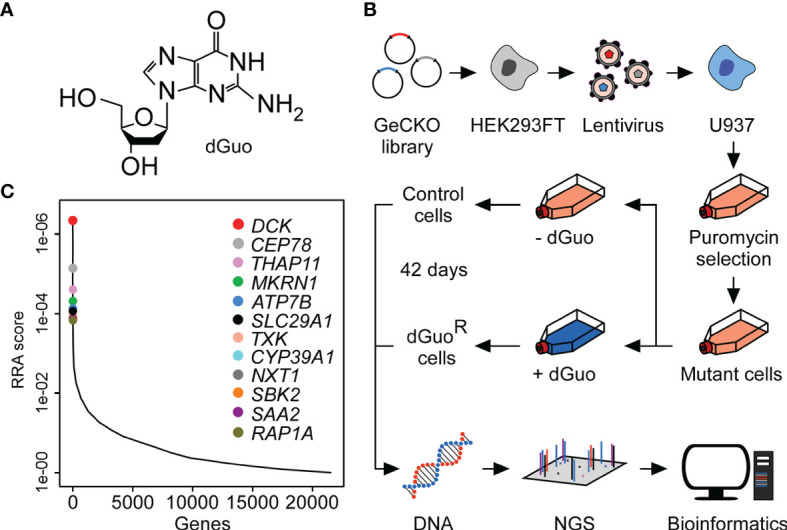
Genome-wide CRISPR-Cas9 screen uncovers host factors conferring susceptibility to cytotoxic deoxyguanosine. **(A)** Chemical structure of deoxyguanosine (dGuo). **(B)** Schematic diagram illustrating the CRISPR-Cas9 screening approach used to identify host determinants that mediate susceptibility of the U937 macrophage cell line to dGuo. **(C)** Discovery of the top candidate genes following dGuo treatment of U937 cells by next generation sequencing (NGS). Data were analyzed using the MaGeCK-based robust rank aggregation (RRA) score analysis and are representative of two independent replicates (see also **Supplementary Figure 3**). A smaller RRA score indicates more essentiality. The twelve top-ranked genes are highlighted.

To discover host factors required for dGuo-mediated cytotoxicity, a genome-wide CRISPR-Cas9 knockout screen was set up with the human macrophage cell line U937. This was achieved by exposing human genome-scale CRISPR-Cas9 knockout (GeCKO) library-transduced U937 cells to a low-dose of dGuo for 42 consecutive days (**Figures 3A, B**). This experimental scheme led to the enrichment of a dGuo-resistant cell population which allowed us to map genes that regulate sensitivity of immune cells to staphylococcal dGuo (**Figure 3C**). Compared to untreated control cells, deep sequencing of the dGuo-resistant cell population along with bioinformatics revealed that dGuo treatment enriched multiple small-guide RNAs (sgRNA) targeting an array of genes, including those encoding for deoxycytidine kinase (*DCK*), centrosomal protein 78 (*CEP78*), thanatos-associated protein 11 (*THAP11*), makorin ring finger protein 1 (*MKRN1*), ATPase copper transporting beta (*ATP7B*), and human equilibrative transporter 1 (hENT1; encoded by *SLC29A1*) (**Figure 3C** and **Supplementary Table 1**). Further, the screen enriched several genes that are associated with cell cycle progression, transcriptional regulation, energy homeostasis, ubiquitination, and apoptosis (**Supplementary Table 1**). In addition, two of the genes enriched most, namely *DCK* and *SLC29A1*, were also identified as top-10 hits in a secondary CRISPR-Cas9 screen indicating that these genes may represent auspicious candidate genes whose products essentially contribute to dGuo-mediated cytotoxicity (**Supplementary Figure 3** and **Supplementary Table 2**). Together, CRISPR-Cas9 screening in U937 cells identified several host factors including the polytopic integral membrane protein hENT1 along with the purine salvage pathway kinase DCK that might play a central role in dGuo-mediated cell death in phagocytes.

### Staphylococcal Deoxyguanosine Targets the Purine Salvage Pathway to Kill Phagocytes *via* Apoptosis

**Figure 4:**
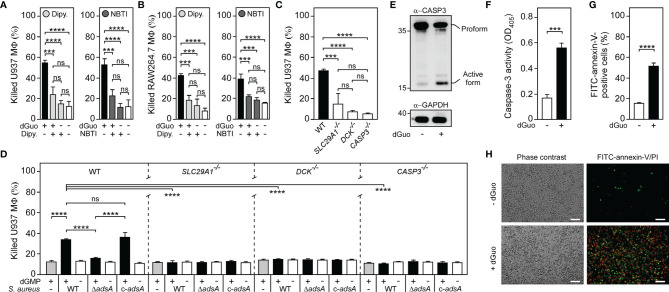
Staphylococcal deoxyguanosine kills macrophages by targeting the purine salvage pathway-apoptosis axis. **(A, B)** Survival of wild-type (WT) U937-derived macrophages (MФ) **(A)** or murine RAW264.7 MФ **(B)** exposed to dGuo in the presence (+) or absence (-) of 10 µM dipyridamole (Dipy.) or nitrobenzylthioinosine (NBTI), both inhibitors of hENT1. Cells were also exposed to the inhibitors only or left untreated. **(C, D)** Survival of WT U937-derived MΦ and their *SLC29A1* ^−/−^, *DCK* ^−/−^, or *CASP3* ^−/−^ variants after treatment with dGuo **(C)** or after treatment culture medium (RPMI 1640) that had been conditioned by incubation with either wild-type (WT) *S. aureus* Newman, its *adsA* mutant (Δ*adsA*), or the complemented *adsA* variant (c-*adsA*) in the presence (black bars) or absence (white bars) of dGMP. Controls included culture medium that had been conditioned by incubation with dGMP only (gray bars) **(D)**. **(E)** Immunoblotting of lysates obtained from dGuo-exposed (+) or untreated (-) WT U937-derived MΦ with caspase-3 and GAPDH-specific antibodies (α-CASP3 and α-GAPDH, respectively). GAPDH was used as a loading control. Numbers to the left of blots indicate the migration of molecular weight markers in kilodaltons. **(F)** Lysates of dGuo-exposed (+) or untreated (-) WT U937-derived MΦ were analyzed for caspase-3 activity using a colorimetric assay. **(G, H)** Analysis of dGuo-dependent induction of apoptosis in WT U937-derived MΦ *via* FACS **(G)** or immunofluorescence microscopy **(H)**. dGuo-exposed (+) or untreated (-) Mϕ were stained using FITC-annexin-V/PI and analyzed as indicated (see also **Supplementary Figure 5**). White bars depict a length of 100 μm. Representative images are shown. 80 µM **(E)**, 160 µM **(A, C, F–H)**, or 320 µM **(B)** of dGuo was used to treat the cells. Cell survival rates were analyzed 24 h **(A, C, D)** or 48 h **(B)** post-treatment. Induction of apoptosis was analyzed 18 h **(F)** or 24 h **(E, G, H)** post-treatment. Data are the mean (± standard deviation [SD]) values from three independent determinations. Statistically significant differences were analyzed with one-way analysis of variance (ANOVA) and Tukey’s multiple-comparison test **(A–D)** or by a two-tailed Student’s t-test **(F, G)** or; ns, not significant (*P* ≥ 0.05); ***P < 0.001; ****P < 0.0001.

Previous work demonstrated that hENT1 transports various nucleosides including staphylococcal dAdo across the cytoplasmic membrane of mammalian cells (29, 48). We surmised that hENT1 may also be responsible for the uptake of cytotoxic dGuo in phagocytes and other dGuo-susceptible cells. If so, hENT1- and DCK-catalyzed activity may therefore lead to the uncontrolled formation of intracellular dGMP, which is further converted to dGDP and dGTP *via* cytosolic guanylate kinase and nucleoside-diphosphate kinase respectively (49–51). To verify this model, we first sought to block hENT1-mediated uptake of dGuo by using two different small molecule inhibitors of hENT1, dipyridamole and nitrobenzylthioinosine (NBTI). Dipyridamole- and NBTI-mediated inhibition of hENT1 transport activity prevented dGuo-induced cell death in U937- or RAW264.7-derived macrophages (**Figures 4A, B**). In agreement with these findings, CRISPR-Cas9-engineered U937-derived macrophages with bi-allelic disruptions in *SLC29A1* (*SLC29A1* ^-/-^) (29) were resistant to both purified or *S. aureus*-derived dGuo confirming that dGuo-mediated cytotoxicity requires hENT1 expression in phagocytes (**Figures 4C, D**). Similarly, U937-derived *DCK* ^-/-^ macrophages (29) were found to be refractory to dGuo-mediated cytotoxicity (**Figure 4C**). Bi-allelic mutations in *DCK* also conferred resistance to staphylococcal dGuo further promoting the idea of dGuo-mediated targeting of the mammalian purine salvage pathway associated with exaggerated biosynthesis of dGTP (**Figure 4D**). In this regard, we note that excessive biogenesis of dGTP following dGuo exposure is a potential apoptotic stimulus in human Jurkat cells (52), raising the possibility that *S. aureus* may synthesize dGuo to trigger apoptotic cell death in phagocytes. To test this conjecture, we exposed wild-type U937-derived macrophages to dGuo and probed cell extracts with a specific antibody that interacts with the inactive pro-form and the cleaved (active) form of human caspase-3, the key modulator of apoptosis. dGuo treatment provoked clipping of pro-caspase-3 to release active caspase-3 (**Figure 4E**). Further, macrophage-derived cell lysates were examined for caspase-3 activity by measuring the hydrolysis of the caspase-3-specific peptide substrate Ac-DEVD-pNA. As expected, dGuo-treatment of U937 phagocytes significantly increased caspase-3 activity suggesting that dGuo-mediated cytotoxicity correlates with apoptotic cell death (**Figure 4F**). Similar findings were obtained for murine RAW264.7 macrophages and primary BMDM further demonstrating that dGuo triggers apoptotic cell death in phagocytes of mammalian origin (**Supplementary Figures 4A, B**). To verify these results, we also exposed U937-derived macrophages to dGuo and assessed dGuo-mediated activation of apoptotic signaling *via* fluorescence-activated cell sorting (FACS) and immunofluorescence microscopy. Specifically, we used a commercially available FITC-annexin-V/PI apoptosis kit which detects both, early apoptotic (that is annexin-V-positive) as well as late apoptotic cells (double-positive for annexin-V and PI). FACS-based analysis of dGuo-exposed macrophages confirmed that dGuo treatment provoked activation of programmed cell death and apoptosis (**Figure 4G** and **Supplementary Figure 5**). Moreover, immunofluorescence microscopy revealed that dGuo treatment triggered apoptosis as positive signals for annexin-V/PI were significantly increased in samples that have been exposed to the drug (**Figure 4H**). Lastly, we took advantage of U937-derived macrophages that lack caspase-3 expression (*CASP3* ^-/-^) (27). When exposed to dGuo or *S. aureus*-derived dGuo, U937-derived *CASP3^−/−^* macrophages exhibited increased resistance to dGuo intoxication in a manner similar to hENT1- or DCK-deficient phagocytes (**Figures 4C, D**). Collectively, these data suggest that *S. aureus* AdsA-derived dGuo targets hENT1 and the purine salvage pathway to promote caspase-3-induced apoptotic cell death in host immune cells.

### Multiplexed Targeting of the Purine Salvage Pathway-Apoptosis Axis Boosts Macrophage Cell Death

**Figure 5:**
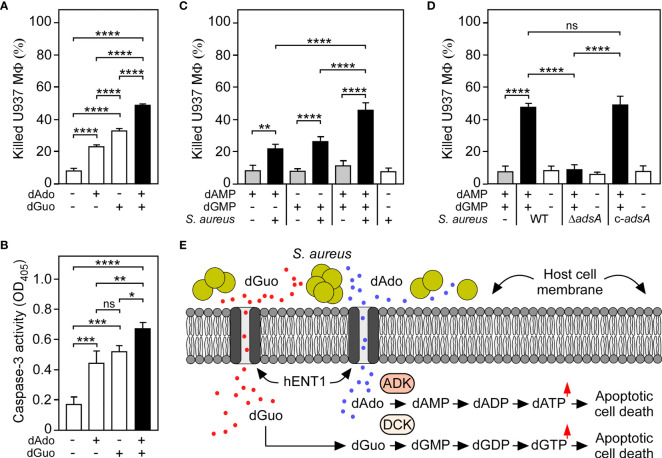
*S. aureus* multiplexes death-effector deoxyribonucleosides to maximize macrophage cell death. **(A)** Survival rates of U937-derived macrophages (MФ) exposed to purine deoxyribonucleosides (dGuo and dAdo) as indicated with + and - symbols. **(B)** Lysates of purine deoxyribonucleoside-exposed (+) or untreated (-) WT U937-derived MΦ were analyzed for caspase-3 activity using a colorimetric assay. Cells were exposed to 160 µM of dGuo or dAdo alone, or received a combination of both **(A, B)**. **(C)** Survival of U937-derived Mϕ after treatment with culture medium (RPMI 1640) that had been conditioned by incubation with wild-type *S. aureus* Newman in the presence (black bars) or absence (white bars) of dGMP and/or dAMP as indicated with + and - symbols. Controls included culture medium that had been conditioned by incubation with dGMP and/or dAMP only (gray bars). **(D)** Survival of U937-derived MΦ after treatment with RPMI 1640 that had been conditioned by incubation with either wild-type (WT) *S. aureus* Newman, its *adsA* mutant (Δ*adsA*), or the complemented *adsA* variant (c-*adsA*) in the presence (black bars) or absence (white bars) of a dGMP/dAMP cocktail. Controls included culture medium that had been conditioned by incubation with a dGMP/dAMP mixture only (gray bars). Cell survival rates or induction of apoptosis were analyzed 18 h **(A, B)** or 24 h **(C, D)** post-treatment. Data are the mean (± standard deviation [SD]) values from three independent determinations. Statistically significant differences were analyzed with one-way analysis of variance (ANOVA) and Tukey’s multiple-comparison test; ns, not significant (*P* ≥ 0.05); *P < 0.05; **P < 0.01; ***P < 0.001; ****P < 0.0001. **(E)** Scheme illustrating *S. aureus*-mediated killing of macrophages *via* synchronous targeting of the purine salvage pathway-apoptosis axis. hENT1 promotes uptake of *S. aureus* Nuc/AdsA-derived purine deoxyribonucleosides into phagocytes. dGuo (red spots) is converted *via* DCK to dGMP, while dAdo-based (blue spots) formation of dAMP is promoted by ADK and DCK. Generation of purine deoxyribonucleoside monophosphates triggers the accumulation of dGTP and dATP, ultimately culminating in the activation of caspase-3 and apoptotic cell death.

Since CRISPR-Cas9 screening uncovered that the underlying principle of dGuo-mediated cytotoxicity is reminiscent of the mechanism previously observed for dAdo (27, 29), we wondered whether *S. aureus* may systematically multiplex purine deoxyribonucleosides in DNA-rich microenvironments to accelerate apoptotic signaling and cell death in macrophages. Particularly, we asked whether simultaneous targeting of the purine salvage pathway-apoptosis axis by AdsA-derived purine deoxyribonucleosides may cause an enhanced cytotoxic effect toward phagocytes. To pursue this possibility, we established a multi-cytotoxicity approach where U937-derived macrophages were exposed to a mixture of dGuo and dAdo, or received each individual drug alone. As expected, single doses of either dGuo or dAdo promoted macrophage cell death (**Figure 5A**). However, a combination of dGuo and dAdo killed significantly more phagocytes in this model, a phenomena that correlated with increased activation of caspase-3 and apoptotic cell death (**Figures 5A, B**). Even lower doses of dGuo and dAdo in the mixture were slightly more potent than higher concentrations of each individual agent alone suggesting that staphylococci may generate a cocktail of cytotoxic purine deoxyribonucleosides for more efficient killing of phagocytes (**Supplementary Figure 6**). To address this further, we next asked whether a combination of *S. aureus*-derived purine deoxyribonucleosides may also enhance immune cell death. To test this, we analyzed survival rates of U937-derived macrophages after treatment with filter-sterilized culture media that had priorly been conditioned by incubation with live staphylococci in the presence or absence of purine deoxyribonucleoside monophosphates. Strikingly, pre-incubation of the AdsA-proficient *S. aureus* Newman wild type strain with a mixture of dGMP and dAMP induced significantly more killing of macrophages in this approach when compared to conditions where staphylococci were incubated with either dGMP or dAMP alone prior intoxication of phagocytes (**Figure 5C**). Moreover, these effects correlated with the expression of *adsA* suggesting that synchronous and AdsA-dependent biogenesis of dGuo and dAdo by *S. aureus*, along with dual targeting of the purine salvage pathway-apoptosis axis, boosts cell death of phagocytes in DNA- or deoxyribonucleotide-rich host environments such as abscesses (**Figures 5D, E**). In summary, these findings indicate that *S. aureus* primarily evolved AdsA for simultaneous and selective biosynthesis of cytotoxic dGuo and dAdo, thereby underscoring the model of deoxyribonucleoside- and AdsA-mediated immune evasion by staphylococci during persistent infections in human or animal hosts.

## Discussion

Deoxyribonucleotides are essential building blocks of nuclear and mitochondrial DNA. In mammalian cells, deoxyribonucleotides are either synthesized by the canonical *de novo* pathway or *via* the nucleoside salvage pathway, which depends on extracellular deoxyribonucleosides and the activity of specific nucleoside transporters as well as rate-limiting deoxyribonucleoside kinases (48, 53). Such intracellular kinases including DCK catalyze the phosphorylation of the respective deoxyribonucleoside resulting in the biogenesis of deoxyribonucleoside monophosphates, which are further converted into deoxyribonucleoside diphosphates and deoxyribonucleoside triphosphates (dNTPs), thereby fueling dNTP pools and the DNA replication machinery in cells that may lack the *de novo* biosynthesis pathway (50, 53).

Beyond the substantial function of dNTPs in all kingdoms of live, maintenance of balanced intracellular dNTP pools is crucial for cell survival and genome integrity. More precisely, cells must tightly regulate the biosynthesis of deoxyribonucleotides as, for example, insufficient supply of precursor molecules or dNTP overload triggers replication errors, proliferation defects, and cell death (53, 54). Intriguingly, *S. aureus* exploits this deadly effect during abscess formation by secreting Nuc and AdsA to synthesize dAdo, which targets the nucleoside salvage pathway to provoke an uncontrolled synthesis of dATP and caspase-3-dependent cell death (26, 27, 29). In this manner, macrophage entry into infectious lesions is markedly limited, thereby boosting pathogen survival in deep-seated abscesses and dissemination of disease (26, 27). However, Nuc-mediated degradation of NETs and DNA released from dying host cells will certainly foster the accumulation of all four canonical deoxyribonucleotides within pyogenic abscesses and purulent exudates, raising the question of whether *S. aureus* may systematically generate a mixture of AdsA-derived effector deoxyribonucleosides to manipulate host nucleotide homeostasis modules and cell death machineries. Consistent with this model, we here demonstrate that the biochemical repertoire of staphylococcal AdsA within DNA-rich microenvironments is not restricted to the biosynthesis of immunomodulatory dAdo but also encompasses excessive biogenesis of dGuo. Of note, dGuo displayed cytotoxic attributes toward monocytes and macrophages of human and animal origin. Similar to the molecular route affecting dAdo-triggered cell death, dGuo-mediated killing of phagocytes involves uptake of dGuo *via* hENT1 and subsequent targeting of the purine salvage pathway to stimulate caspase-3-dependent cell death (**Figure 5E**). Given that dGuo exposure of human cells along with the cascaded activity of DCK, guanylate kinase, and nucleoside-diphosphate kinase correlates with the accumulation of intracellular dGTP (49–51, 55–57), we surmise that staphylococci synthesize dGuo to manipulate intracellular deoxyribonucleotide pools of macrophages, ultimately culminating in the activation of the apoptotic signaling cascade to promote a rapid and immunologically silent type of cell death. In this context, we further note that not only phagocytes but also lymphocytes exposed to dGuo exhibit apoptotic features suggesting that staphylococci eventually produce dGuo to perturb adaptive immune cell responses and immune cell networking during acute infection (52, 58, 59). However, neutrophils appear less susceptible to dGuo-mediated cytotoxicity, probably as a result of differentially regulated host determinants or cellular immunity factors that directly affect dGuo-triggered cell death. For example, SAMHD1, a dNTP-degrading phosphohydrolase and intracellular enzyme that restricts HIV-1 infection of myeloid cells (60), along with the purine nucleoside phosphorylase PNP affect survival rates of dGuo-exposed cells (58, 59, 61, 62). Altered expression levels of the appropriate genes or genetic elements of the purine salvage pathway, together with limited uptake capacity of hENT1 in less-susceptible cells, may therefore explain the specific cytotoxicity tropism of dGuo. Further, single nucleotide polymorphisms (SNPs) in candidate genes may change the susceptibility pattern of host cells toward dGuo. Specifically, various functional SNPs such as rs67437265 (c.364C→T; p.Pro122Ser) have been identified in the DCK-encoding gene that presumably hamper dGuo-mediated intoxication of phagocytes (63). If so, such genetic variants may influence abscess development and eventually account for variable clinical outcomes of hospitalized patients and specific ethnic groups which display altered vulnerability to *S. aureus* infections (9). Nevertheless, the analysis of the enzymatic properties of AdsA revealed further that this enzyme predominantly contributes to the biogenesis of purine deoxyribonucleosides (i.e. dGuo and dAdo) in DNA-rich microenvironments, a fact that is of paramount importance as purine deoxyribonucleosides typically exhibit more cytotoxic potential toward mammalian cells than pyrimidine deoxyribonucleosides (55, 64). In this regard, it is tempting to speculate that *S. aureus* may selectively synthesize purine deoxyribonucleosides to accelerate killing of phagocytes and other innate immune cells. Indeed, synchronous biogenesis of dGuo and dAdo, together with multiplexed targeting of the purine salvage pathway-apoptosis axis maximized cell death of macrophages and may therefore tune *S. aureus* intra-abscess survival and bacterial spread in diseased hosts. Concurrently, the advanced biochemical properties of AdsA could help the pathogen to secure the continued biogenesis of at least one cytotoxic deoxyribonucleoside during infection as the complex microenvironment of abscesses and infectious foci along with the variable GC-content of mammalian DNA fragments may locally restrict access of AdsA to appropriate and sufficient substrates.

Overall, we propose that *S. aureus* evolved AdsA for the excessive biogenesis of dGuo and dAdo, two cytotoxic purine deoxyribonucleosides that simultaneously target the nucleoside salvage pathway-apoptosis axis to promote immune cell death. Following this two-pronged strategy, staphylococci effectively block macrophage access to the bacterial abscess community located in the core of mature lesions without causing traitorous inflammatory signals, ultimately shaping the pathological architecture of abscesses in the skin or organ tissues. Since AdsA is also catalyzing the biosynthesis of immunosuppressive adenosine in blood (31), and homologues of AdsA were previously identified in other clinically relevant Gram-positive microbes including emerging methicillin-resistant *Staphylococcus pseudintermedius*, *Bacillus anthracis*, and several pathogenic streptococci (31, 38, 65–69), development of small molecule inhibitors or therapeutic monoclonal antibodies that neutralize AdsA may help to combat infections caused by MRSA and other drug-resistant bacterial pathogens.

## Data Availability Statement

The original contributions presented in the study are included in the article/**Supplementary Material**, further inquiries can be directed to the corresponding author. Raw sequence data for the CRISPR-Cas9 screen are available at European Nucleotide Archive (ENA) (PRJEB50054).

## Ethics Statement

Human blood samples were obtained from anonymous, consenting healthy donors from the Hannover Medical School blood donation center. The Hannover Medical School blood donation center obtained written informed consent from all participants involved in the study. These studies were reviewed and approved by the medical ethics committee of Hannover Medical School (Hannover, Germany) under the permission number 8831_BO_K_2019. Scientific use of animals was carried out under approved animal care and use protocols which are granted by the state veterinary authorities and overseen by the internal animal care and use committee (IACUC).

## Author Contributions

VW conceived the project. ET, NS, and VW designed and planned the experiments. ET, NS, TO, AG, HB, MV, and VW performed the experiments. ET, NS, TO, AG, HB, MV, and VW analyzed the results. VW wrote the paper. ET, NS, TO, AG, HB, MV, and VW provided revisions. All authors substantially contributed to the article, and approved the submitted version.

## Funding

Work in the VW laboratory is supported by the German Research Foundation (award WI4582/2-1 to VW; project number 449712894) and the Else Kröner-Fresenius-Stiftung (award 2021_EKEA.16 to VW). ET and NS were supported by the Hannover Biomedical Research School (HBRS) and the Center for Infection Biology (ZIB).

## Conflict of Interest

The authors declare that the research was conducted in the absence of any commercial or financial relationships that could be construed as a potential conflict of interest.

## Publisher’s Note

All claims expressed in this article are solely those of the authors and do not necessarily represent those of their affiliated organizations, or those of the publisher, the editors and the reviewers. Any product that may be evaluated in this article, or claim that may be made by its manufacturer, is not guaranteed or endorsed by the publisher.
