# Supplementary material for: Staphylococcus aureus Multiplexes Death-Effector Deoxyribonucleosides to Neutralize Phagocytes
Source: Front Immunol. 2022 Mar 10;13:847171. doi: 10.3389/fimmu.2022.847171 (PMC8960049; doi:10.3389/fimmu.2022.847171)
Supplement: Supplementary file 1 [file DataSheet_1.pdf]

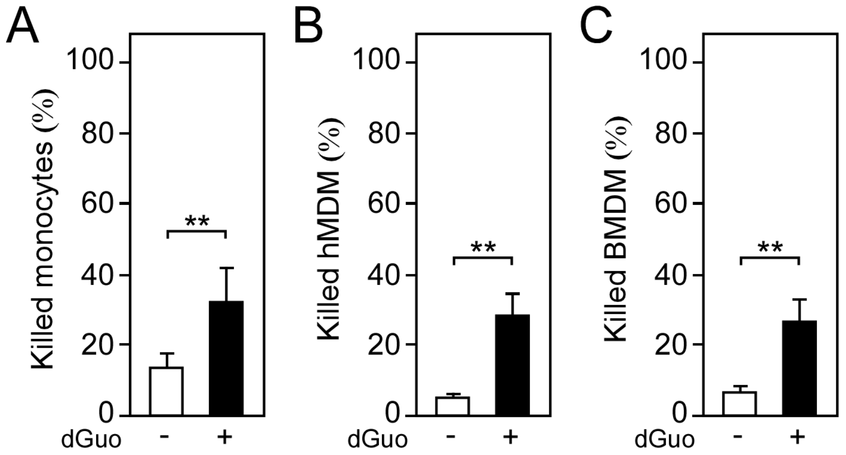

Supplementary Figure 1. Deoxyguanosine promotes killing of human and murine primary cells. (A-C) Survival rates of primary human CD14$^+$ monocytes (A), human monocyte-derived macrophages (hMDM) (B), or murine bone marrow-derived macrophages (BMDM) (C) exposed to dGuo (+) or left untreated (-). Cells were treated with 160 µM (CD14$^+$ monocytes; BMDM) or 320 µM (hMDM) of dGuo. Cell survival rates were analyzed 24 h (panels A, B) or 72 h (panel C) post-treatment. Data are the mean (± standard deviation [SD]) values from at least three independent determinations. Primary cell experiments include at least three independent donors. Statistically significant differences were analyzed by a two-tailed Student's t-test; **, $P < 0.01$.

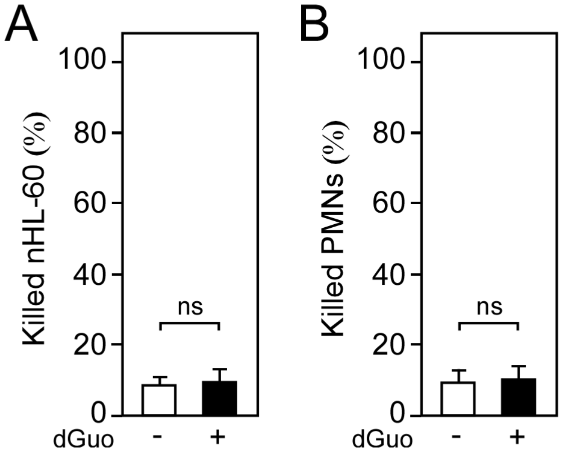

Supplementary Figure 2. Neutrophils are resistant to deoxyguanosine. (A, B) Survival rates of neutrophil-like HL-60 cells (nHL-60) (A) or primary human neutrophils (PMNs) (B) exposed to dGuo (+) or left untreated (-). Cells were treated with 160 µM dGuo. Cell survival rates were analyzed 24 h post-treatment. Data are the mean (± standard deviation [SD]) values from three independent determinations. Primary cell experiments include three independent donors. Statistically significant differences were analyzed by a two-tailed Student's t-test; ns, not significant ($P \geq 0.05$).

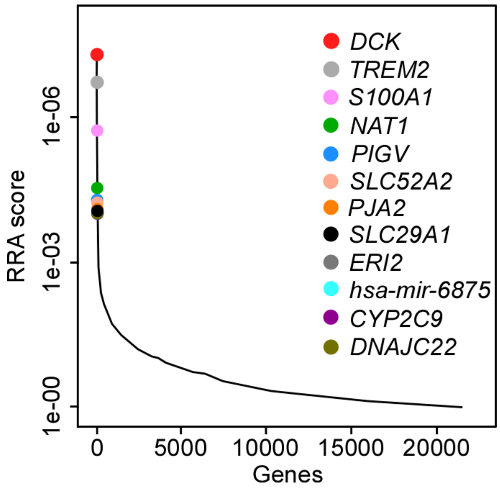

Supplementary Figure 3. Independent CRISPR-Cas9 screen discovers host determinants conferring susceptibility to cytotoxic deoxyguanosine. Results of an independent genome-wide CRISPR-Cas9 screen used to identify host factors that mediate susceptibility of the U937 macrophage cell line to deoxyguanosine (dGuo). Top candidate genes following dGuo treatment of U937 cells were identified via next generation sequencing. Data were analyzed using the MaGeCK-based robust rank aggregation (RRA) score analysis. A smaller RRA score indicates more essentiality. The twelve top-ranked genes are highlighted.

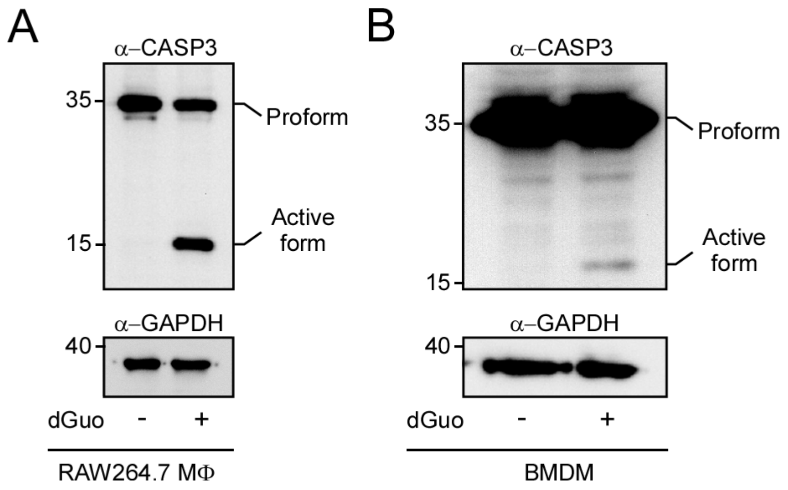

Supplementary Figure 4. Cleavage of caspase-3 is required for deoxyguanosine-triggered cell death in murine macrophages. (A, B) Immunoblotting of lysates obtained from dGuo-exposed (+) or untreated (-) murine RAW264.7 MΦ (A) or bone marrow-derived macrophages (BMDM) (B) with caspase-3 and GAPDH-specific antibodies (α-CASP3 and α-GAPDH, respectively). GAPDH was used as a loading control. Numbers to the left of blots indicate the migration of molecular weight markers in kilodaltons. 320 µM of dGuo was used to treat the cells. Cell lysates were analyzed 24 h post-treatment. Representative images are shown.

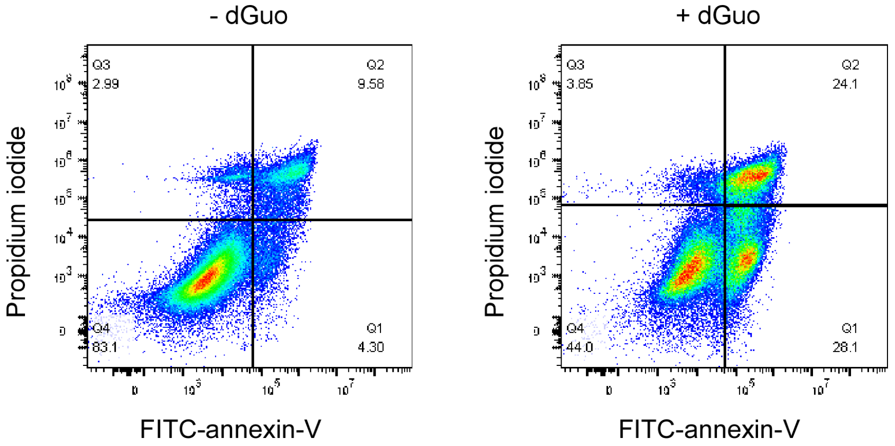

Supplementary Figure 5. Deoxyguanosine induces apoptosis in human macrophages. Representative FACS plots of human U937-derived macrophages (MΦ) stained with FITC-annexin-V/PI following exposure to dGuo (160 µM) for 24 h.

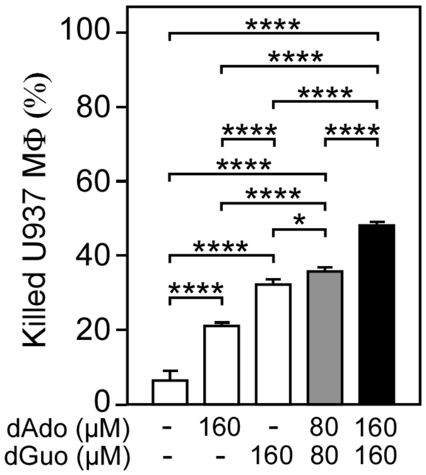

Supplementary Figure 6. Co-treatment of human macrophages with purine deoxyribonucleosides promotes cell death. Survival rates of U937-derived macrophages (MΦ) exposed to various concentrations of purine deoxyribonucleosides (dGuo and dAdo). Cells were exposed to dGuo or dAdo alone, or received a combination of both. Cell survival rates were analyzed 18 h post-treatment. Data are the mean (± standard deviation [SD]) values from three independent determinations. Statistically significant differences were analyzed with one-way analysis of variance (ANOVA) and Tukey's multiple-comparison test; *, $P < 0.05$; ****, $P < 0.0001$.
